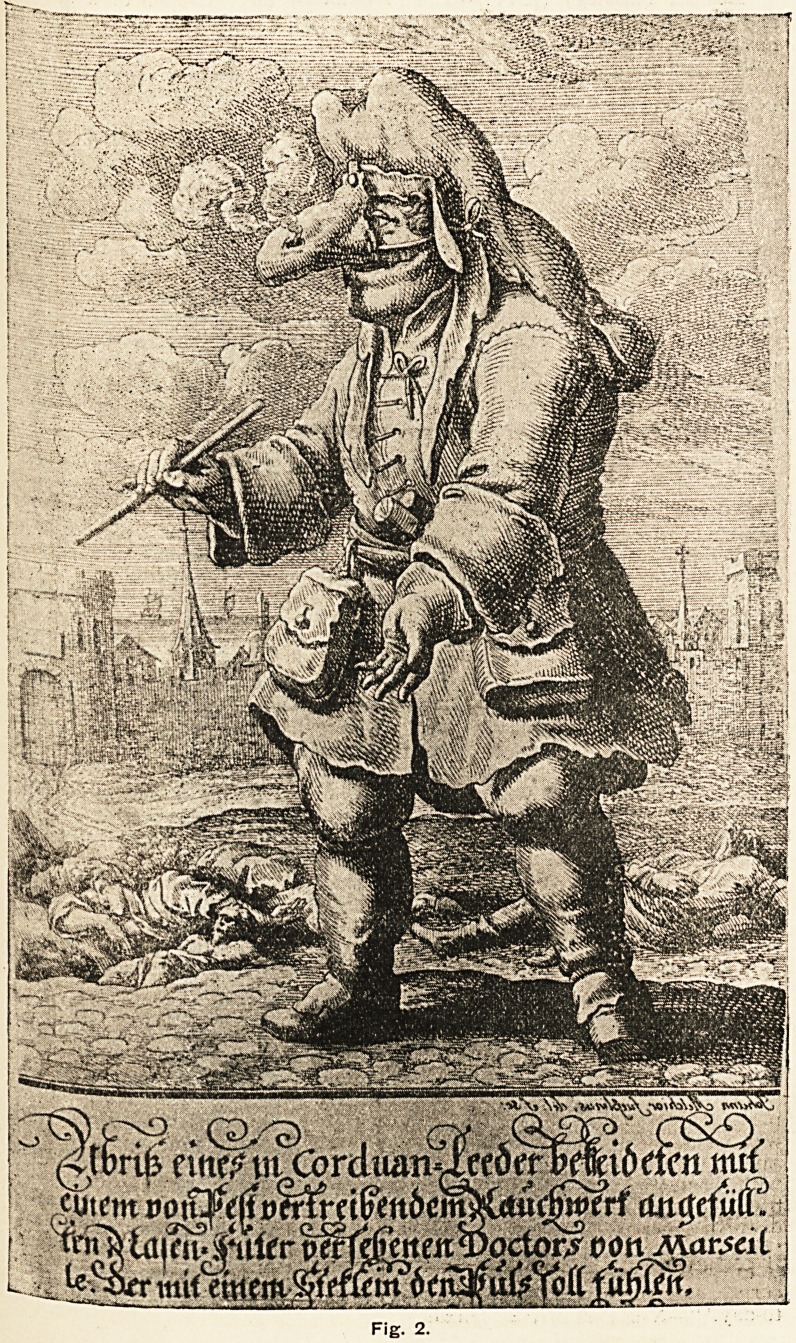# Scraps

**Published:** 1898-03

**Authors:** 


### SCRAPS

PICKED UP BY THE ASSISTANT-EDITOR.

**Clinical Records** (22).—Convalescent (dictating): "Please say to Mrs. Jackson that I thank her not alone for the brandy peaches that she so kindly sent me, but for the spirit in which they were sent."—*Journal of the American Medical Association.*

**Sanitary Science.**—As we have lately had prominently before us the subject of zymotic disease, it is appropriate to quote from an article in the *Sanitary Record*, in which Mr. H. Percy Boulnois records some answers which were given at examinations at the Sanitary Institute in the unenlightened days before candidates for sanitary inspectorships had the opportunity of bringing themselves to the high degree of perfection they have since attained.

One candidate, speaking of small-pox, said: "Small-pox is very 'catchy,' I've known cases spread over half a mile." And as to precautions to be taken with regard to the prevention of the spread of disease, the answer was given that "All discharges from the body of a cholera patient should be given to the Sanitary Authority to dispose of." Speaking of the necessary precautions to be taken during a severe case of small-pox, the answer was, "A precaution to be taken in small-pox is that the patient should be given an old rag for his own use," and another was of opinion that it was sufficient "to inquire whether the house was overcrowded and properly ventilated." Why the necessity for this summary proceeding does not readily appear, though for thoroughgoing obscurity of meaning the following answer takes the palm: "Cholera spreads through the air; as a proof of it, if you fly a kite with a piece of meat attached it turns black. Also by flying lights." Another answer with which, though we may thoroughly agree, we are somewhat puzzled, is that "No person should either give, use, or lend typhoid fever;" and another answer which occurred more than once at a provincial examination rather puzzled the examiners, as it looked as if the candidates must have been coached in some way, was that "Infected excreta must be stirred with a red-hot poker, taking care to bring the whole of it in contact." A rather unpleasant and dangerous operation for the nurse or inspector, if it formed part of his duty, and we may even infer the following suggestion as to dealing with infected emanations, though the wording is suggestive of a different meaning from that no doubt intended to be conveyed by the candidate: "Excreta must be covered with chloride of lime and taken in the night;" and a rather curious answer to a question as to what was meant by a covered receptacle, was that "It was one covered by carbolic acid."

In reply to another question as to "What is commercial carbolic acid and what is it used for?" a candidate gave the following elaborate and remarkable answer: "Commercial carbolic acid is used by commercial people on their entering different dwellings in their travelling intercourse with the public, especially for their safety in sleeping apartments in different hotels and lodging-houses." This reply is considerably wide of the mark, as was that on a question of ventilation, when the reply was, "Oh, ventilation goes on by assumption;" and also the answer as to the objections to damp houses, which ran as follows: "Dry houses are more cheerful, as in damp ones the inmates must be continually mourning the loss of something in the shape of a death."

In the case of death arising from an infectious disease, the candidate said that "If the *body* or friends of deceased wish to prolong burial they must obtain a certificate from the Medical Officer of Health;" and another said that "It shall not be lawful to allow any body that has died of infectious disease in any bed-room, workshop," &c. What was to be done with it does not transpire; we can only trust it was not the same candidate who, in reply to a question as to what precautions he should take after a case of infectious disease, said he should "at once *throw everything out of the window*." We recommend the following answer to these candidates: "Where a person having died of an infectious disease *is without proper lodging accommodation*, the Local Authority may, by order of the justice, empower any of their officers to remove such person to the mortuary."

**Medical "Bits of Old Bristol"** (6).—I have not been able to find any account of special preventive measures adopted in Bristol during the Black Death epidemic of 1348. Probably the course of proceedings was much the same as that which Boccaccio in the Introduction to the *Decameron* describes as having taken place in Florence. In the Sydenham Society volume, entitled *The Epidemics of the Middle Ages*—a work which consists of a translation by Babington of Hecker's "Der Schwarze Tod," "Die Tanzwuth," and "Der Englische Schweiss"—Boccaccio is quoted as saying that some of the inhabitants "shut themselves up in their houses, with their wives, their children and households, living on the most costly foods, but carefully avoiding all excess. None were allowed access to them; no intelligence of death or sickness was permitted to reach their ears; and they spent their time in singing and music, and other pastimes. Others, on the contrary, considered eating and drinking to excess, amusements of all descriptions, the indulgence of every gratification, and an indifference to what was passing around them, as the best medicine, and acted accordingly. They wandered day and night from one tavern to another, and feasted without moderation or bounds. In this way they endeavoured to avoid all contact with the sick, and abandoned their houses and property to chance, like men whose death-knell had already tolled." Others "ate and drank what they pleased, and walked abroad carrying odoriferous flowers, herbs or spices, which they smelt to from time to time, in order to invigorate the brain, and to avert the baneful influence of the air infected by the sick, and by the innumerable corpses of those who had died of the plague. Others carried their precaution still further, and thought the surest way to escape death was by flight. They therefore left the city; women as well as men abandoning their dwellings, and their relations, and retiring into the country." (See also Wright's edition of the *Decameron* [1872], Chatto and Windus, pp. 30–31.) Almost all the physicians of the time held that the disease originated from astrological influences, yet they of course recognised that it was propagated by contagion. But the directions for preventing its spread consisted for the most part, as may be seen from the pronouncement of the College of Physicians of Paris, of minute and impracticable instructions about diet. I hope in a future number to give a few notes concerning the water-supply of Bristol at the period.

At the time of this invasion of the pestilence public opinion was not ripe for the adoption of severe measures of isolation, and little more of a general character was done than the attempted purification of the air by means of large fires in the affected districts. But before the century ended harsh methods were adopted, the nature of which may be gathered from the statement of Hecker (*Op. cit.*, p. 62), who says the regulation issued in Italy in 1374 ordained that every plague-patient was to be taken out of the city into the fields, there to die or to recover, and that those who attended upon a plague-patient were to remain apart for ten days, before they again associated with any body; that whoever imported the plague, the State condemned his goods to confiscation, and that none except those who were appointed for that purpose, were to attend plague-patients, under penalty of death and confiscation. We read that in 1399 infected houses were to be ventilated for at least eight or ten days, and purified from noxious vapours by fires, and by fumigations with balsamic and aromatic substances. Straw, rags, and the like, were to be burned; and the bedsteads which had been used, set out for four days in the rain or the sunshine, so that, by means of the one or the other, the morbific vapour might be destroyed. No one was to venture to make use of clothes or beds out of infected dwellings, unless they had been previously washed and dried, either at the fire or in the sun. By 1485 at Venice lazarettos were established of two kinds. In one the sick were treated, and when apparently well were detained for forty days in the other. The period of forty days from which we get the term quarantine was adopted partly on account of its frequent mention in the Bible as a term of isolation, and partly in deference to medical dogmatism, which had decreed forty days to be a critical time in many acute diseases, and had divided the period of gestation into seven such epochs which were supposed to mark definite developments of the fœtus.

For a long time arsenic worn as an amulet had a great reputation as a prophylactic. "W. Kemp, Mr. of Arts," who in 1665 issued *A Brief Treatise of the Nature, Causes, Signes, Preservation from, and Cure of the Pestilence*, speaks highly of several formulæ devised for the purpose. He cites with special commendation one from Rhenanus which was prepared by the following method: "Take of white and yellow Arsenick of each half an ounce, the powder of dried Toads two ounces, Mercury sublimed, Wheat Flowre, the Roots of Dittany, of each three drams, Saffron, the Fragments of Jacynth and Emerald of each one scruple, make them all into powder, and with Gum Dragon dissolved in Rose-water, make them into Cakes." These, which were to be "about the breadth of a Shilling and the thickness of two half Crowns," were to be dried in the sun or in an oven after the bread was taken out. Kemp directs his readers to "sew them in a little silk bag, fastening it to a ribbon, and hanging it about your Neck, let it lie about the middle of your Breast," and adds, "I have known some of these worn in the City of Bristol, in the time of the Plague, and the parties sometimes would have little pimples like the Itch, rise about the breadth of the Amulet in their Breast, which they did rub and scratch, but never had the Plague, and are alive till now."

It would be interesting if it could be discovered that Bristol doctors at any time took individual precautions at all like those which are mentioned in *Janus*, 1896–97, i. 97–103. Dr. Ch. Fiessinger there calls attention to the sanitary regulations employed in the 17th century to prevent the spread of the plague, and he reproduces from Manget's *Traité de la Peste*, published in Geneva in 1721, the drawing of the dress (Fig. 1) which should be worn by those visiting the plague-stricken. Manget says: "L'Habit exprimé dans cette figure n'est pas une chose de nouvelle invention, & dont on ait commencé l'usage dans la dernière Peste de Marseille: Il est d'une plus vielle datte (*sic*), & Messieurs les Italiens ont fourni à peu près de semblables figures, depuis fort longues annèes. Le nès en forme de bec, rempli de purfums (*sic*) & oint intérieurement de matiéres balsamiques, n'a véritablement que deux trous, un de chaque côté, à l'endroit des ouvertures du nès naturel; mais cela peut suffire pour la respiration, & pour porter avec l'air que l'on respire l'impression des drogues renfermées plus avant dans le bec. Sous le Manteau, on porte ordinairement des Bottines à peu près à la Polonaise, faites de Maroquin de Levant; des Culottes de Peau unie, qui s'attachent aux dites Bottines; & une Chemisette aussi de Peau unie, dont on renferme le Bas dans les Culottes; le Chapeau & les Gans sont aussi de même Peau."

Alluding to Manget's statement that the dress was not a recent device, M. Reber, in a later number of *Janus*, pp. 297–300, refers to an article in *La Revue de Paris*, 1896, pp. 191–218, in which is quoted a description of a somewhat similar dress that Dr. Charles de L'Orme, physician to Louis XIII., adopted during a visitation of the plague in 1619. M. Reber possesses a drawing (Fig. 2) which represents the dress worn by a Marseilles doctor during a similar epidemic. The artist was Jean Melchior Fuesslinus, who lived from 1677 to 1736. The description in German is: "Abriss eines in Corduan-Leeder bekleideten mit einem von Pest-vertreibendem Rauchwerk angefüllten Nasen-Futer versehenen Doctors von Marseille. Der mit einem Steklein den Puls soll fühlen," which in English would read: "Sketch of a Doctor of Marseilles dressed in Cordovan leather and provided with a nose-case filled with a fumigating substance for keeping off the pestilence. He is to feel the pulse with a wand." The two figures[1] therefore represent the dress of practically the same period. The stick in the hand of Manget's figure seems too substantial to serve the purpose of feeling the pulse of the patient for which the lighter rod carried by the Marseilles doctor was intended.

Fig. 1.

1 For the loan of the blocks of these pictures I am indebted to the kindness of Dr. H. F. A. Peypers, the courteous editor of *Janus*.

Abriß eines in Corduan-Leeder bekleideten mit einem von Pest vertreibendem Rauchwerk angefüllten Nasen-Futer versehenen Doctors von Marseil le. Der mit einem Steklein den Puls soll fühlen.

Fig. 2.

**Medical Philology** (XXV.)—The *Promptorium* has "Cropōn of a beste," and the *Catholicon* has "a Croypon̄." The Latin equivalent in each case is *clunis*. Mr. Herrtage's note is:

"'*Clunis*. The buttock or hanche.' Cooper. '*Cropion*. The rump or crupper. *Le mal de cropion*. The rumpe-evill or crupper-evill; a disease wherewith small (cage) birds are often troubled.' Cotgrave."

H. G. Adams, in *Cage and Singing Birds*, says that the rump gland "forms part of the structural economy of every bird, and is intended for secreting the oily substance required to render the plumage supple and impervious to wet. The bird presses this gland, which is situated just above the rump, with its bill, and the oil oozes out; if this is not done frequently, the opening is apt to get clogged, and there being no vent for the increasing contents of the gland, it gets hard and inflamed."

The *New English Dictionary* gives several instances of the word *crupper*, popularly applied to that portion of the body which, because it "has been generously supplied by Providence with some sixteen different sensory nerves," is, in the opinion of a recent writer in the *Lancet*, specially marked out in bad boys for the application of a birch rod. The word is, however, not unknown in scientific anatomy, for the Sydenham Society Lexicon and Dr. Gould's Dictionary both give "crupper-bone, the coccyx."

**A Physician's Estimate of his Class.**—In Dr. S. Weir Mitchell's interesting "medicated novel," *Characteristics*, there is the following description of varieties of medical men that will suggest acquaintances to many of our readers: "There is no place where good breeding has so sweet a chance as at the bedside. There are many substitutes, but the sick man is a shrewd detective, and soon or late gets at the true man inside of the doctor.

"I know, alas! of men who possess cheap manufactured manners adapted, as they believe, to the wants of 'the sick-room'—a term I loathe. According to the man and his temperament do these manners vary, and represent sympathetic cheerfulness or sympathetic gloom. They have, I know, their successes and their commercial value, and may be of such skilful make as to deceive for a time even clever women, which is saying a great deal for the manufacturer. Then comes the rarer man who is naturally tender in his contact with the sick, and who is by good fortune full of educated tact. He has the dramatic quality of instinctive sympathy, and, above all, knows how to control it. If he has directness of character too, although he may make mistakes (as who does not?), he will be, on the whole, the best adviser for the sick, and the completeness of his values will depend upon mental qualities which he may or may not possess in large amount.

"But over and above all this there is, as I have urged, some mystery in the way in which certain men refresh the patient with their presence. I fancy that every doctor who has this power—and sooner or later he is sure to know that he has it—also learns that there are days when he has it not. It is in part a question of his own physical state; at times the virtue has gone out of him.

"I had a rather grim but most able surgeon. He seemed to me to have a death-certificate ready in his pocket. He came, asked questions, examined me as if I were a machine, and was too absorbed in the *physical me* to think about that *other me*, whose tentacula he knocked about without mercy, or without knowledge that tenderness was needed. Our consultant was a physician with acquired manners. He always agreed with what I said, and was what I call aggressively gentle; so that he seemed to me to be ever saying with calm self-approval, 'See how gentle I am.' I am told that with women he was delightfully positive, and I think this may have been true, but he was incapable of being firm with the obstinate. His formulas distressed me, and were many. He was apt to say as he entered my room, 'Well, and how are we to-day?' And this I hated, because I once knew a sallow undertaker who, in the same fashion, used to associate himself with the corpse, and comfort the living with the phrase, 'We are looking quite natural to-day.'"—*New York Medical Journal*.

## Figures and Tables

**Fig. 1. f1:**
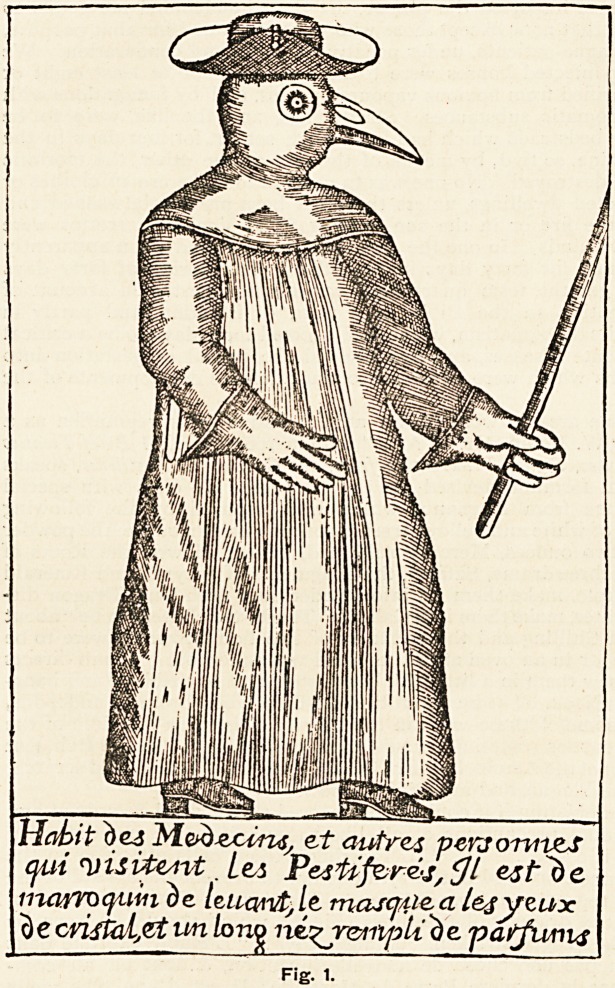


**Fig. 2. f2:**